# Design and Analysis of Dual-Polarized Frequency-Selective Metasurface for X-Band Notch Applications

**DOI:** 10.3390/s26030867

**Published:** 2026-01-28

**Authors:** Muhammad Idrees, Sai-Wai Wong, Yejun He

**Affiliations:** State Key Laboratory of Radio Frequency Heterogeneous Integration, Sino-British Antennas and Propagation Joint Laboratory of MOST, Guangdong Engineering Research Center of Base Station Antennas and Propagation, Shenzhen Key Laboratory of Antennas and Propagation, College of Electronics and Information Engineering, Shenzhen University, Shenzhen 518060, China; muidrees169@gmail.com (M.I.); yjhe@szu.edu.cn (Y.H.)

**Keywords:** angle of incidence, electromagnetic interference (EMI), frequency-selective metasurface, polarization-insensitive, selective shielding, X-band

## Abstract

This paper presents a miniaturized, polarization-insensitive frequency-selective metasurface (FSMS) with stopband behavior for RF shielding applications. The FSMS is designed to suppress communication at 10 GHz frequency in the X-band. The design comprises a circular metallic patch with a staircase slot engraved in the center. The FSMS achieves an attenuation of 38.5 dB at the resonant frequency with a 10 dB suppression fractional bandwidth of more than 46%. The physical geometry of the unit cell makes it polarization-independent, and the angle of incidence has no effect on the stopband. The FSMS cell has overall dimensions of 0.3*λ_o_* × 0.3*λ_o_* × 0.05*λ_o_*, where *λ_o_* is free-space wavelength at the resonant frequency. Moreover, an equivalent circuit model (ECM) of the FSMS filter is developed to analyze its operation principle. An FSMS prototype is fabricated and tested for its performance, and the simulated and measured results show good agreement, making it suitable for selective electromagnetic interference (EMI) shielding applications.

## 1. Introduction

The unprecedented developments in the communication industry have caused a proliferation of smart devices, communication systems, and intelligent warfare equipment, which have influenced daily routines. Although this growth has revolutionized connectivity, it also brings significant challenges, like electromagnetic interference (EMI). EMI has become an inevitable issue that affects communication, malfunctioning, and performance degradation of nearby electronic gadgets. To address this challenge, EM absorbers, wire meshing, metallic screens and enclosures, etc., are typically used to reduce unwanted radiation [1]. These techniques are effective for general-purpose applications while having limitations, i.e., they might block all transmissions, bulkiness, and cost, making them ineffective for modern applications where selective shielding is required. However, frequency-selective metasurfaces offer better EMI-selective shielding solutions due to their versatile stable spectral responses, small size, low cost, easy manufacturing, and employability.

Frequency-selective metasurfaces (FSMSs) are capable of manipulating the EM spatial waves, revealing either reflection or transmission characteristics as a function of frequency [2]. FSMSs have attracted overwhelming research attention due to their employability in fields like antenna radome design [3], RCS reduction [4,5], EM absorbers [5,6,7], reflectors [8], polarization rotating surfaces [9], antenna design [10,11], space and satellite communications [12,13], reflectarray [14], square kilometer array designs [15], BIC metasurface-based 3D imaging systems [16], EMI shielding [17,18], and others.

Many recent works have focused on designing FSSs with band-reject characteristics to meet specific requirements. A compact interdigitated loop elements-based FSS shield in [19], a convoluted loop element [20], and a miniaturized modified swastika cell in [21] are reported for various shielding applications. However, these FSS designs have performance constraints for transverse magnetic (TM) polarization mode. A reflective FSS in [22] accomplishes linear-to-circular polarization conversion. In [23], a miniaturized convoluted conducting element printed over an FR-4 substrate effectively shields EM waves at 10 GHz, featuring dual operation modes and polarization-independent performance. A mechanically tunable FSS-based venetian blind for indoor window shielding is presented in [24]. In [25], single-layer FSS effectively isolates satellite downlink frequencies but it reveals dissimilar response for TE mode and TM modes. In [26], a square ring element was applied to various curved surfaces to investigate its effective shielding characteristics in the X-band (8–12 GHz).

Moreover, a study in [27] reports a pair of rhombic loops attaining a stopband response to suppress X-band signals, yet it has limited angular stability up to 45°. An FSS comprising two patch layers provides near-field shielding in the X-band with a 10 dB suppression bandwidth of 32% [28]. Reference [29] introduces a low-profile dual-element EM shield suppressing interference while ensuring angularly stable and polarization-insensitive spectral responses. A compact symmetrically modified square ring structure [30] is designed for rejection of the ultra-wideband (UWB) frequency range. In addition, studies [14,31,32,33] employ identical or different resonating structures printed on a dielectric, leveraging the mutual coupling effect between parallel patches on opposite sides to accomplish wideband and ultra-wideband shielding characteristics as well as unit cell miniaturization. A strong coupled FSS structure in [34] achieves miniaturized cell size with higher angular stability and polarization insensitivity. However, its rejection bandwidth and attenuation level decrease with the incident angle for the TM polarization mode.

Most of the above structures have performance constraints regarding suppression of the bandwidth and shielding effectiveness (SE) when illuminated by TM-polarized waves. This article aims to present a compact, dual-polarized FSMS with band-notch characteristics for EMI mitigation. The FSMS comprises a circular metallic patch with a symmetric staircase profile, printed on a lossy substrate. The stepped profile contributes to achieve unit cell miniaturization, wide-bandwidth, and improves angular stability compared to a traditional cross-slot design. The FSMS efficiently reflects the X-band signals with the following silent features. The main contributions of this paper are as follows.

- The FSMS features a single-layer simple structure, easy fabrication and installation, as well as providing a cost-effective solution for alleviating unwanted interference.
- It accomplishes SE of at least 38.5 dB at 10 GHz X-band frequency with a 10 dB fractional rejection bandwidth of 46% at normal incidence for vertical and horizontal polarizations. It is also scalable to other frequencies.
- The design offers wide angular stability up to 70° for un-rotated and rotated configurations, revealing polarization-insensitive spectral characteristics under both the TE and TM wave modes. It mitigates degradation effects in SE and stop-bandwidth with incident angle specifically for the TM mode, which is a challenge in many existing FSS designs [19,20,25,27,28,35,36].
- FSMS confirms complete polarization independence and being rotation-independent as an additional feature, making it appropriate for selective shielding applications.

The rest of this paper is organized as follows: Section 2 presents design description of FSMS. Section 3 describes simulated results and optimization of shielding effectiveness, along with an equivalent circuit model. Section 4 specifies a detailed parametric analysis. The measurement setup and measured results are discussed in Section 5. Finally, Section 7 summarizes and concludes the paper.

## 2. FSMS Design Topology

**Figure 1:**
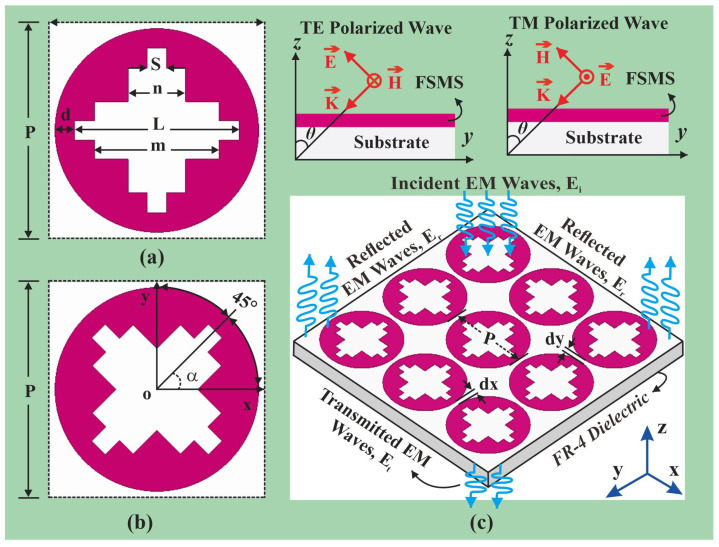
Schematic view of the proposed FSMS. (**a**) Labeled top view of the FSMS radiation element, (**b**) FSMS element rotated at 45°, (**c**) a 3 × 3 element FSMS array rejecting the X-band signals while being transparent to rest of the frequencies. While optimized design variables are d=0.8 mm, L=6.9 mm, m=5.2 mm, n=2.4 mm, P=9 mm, S=0.8 mm, $d_{x}=d_{y}=0.5$ mm, *α* =45°.

The unit cell of the FSMS is composed of a circular metallic patch with a staircase slot in the center, as depicted in Figure 1a. The circular metallic patch has a diameter of L+2d, designed on an FR-4 substrate having a thickness of 1.55 mm, a relative permittivity of $\varepsilon_{r}$ = 4.4, and a dielectric loss tangent (tanδ) of 0.02. A staircase slot is introduced in the FSMS unit cell. The staircase is designed by subtracting rectangular strips from the circular metallic patch. These subtracting strips have different lengths, but the same width of 0.8 mm. The central horizontal portion of the slot has an area of L×S with two accompanying slots on the top and bottom with an area of m×n each. The same geometry is replicated in the vertical direction. The staircase profile serves to achieve the desired maximum transmission loss. Moreover, the staircase profile provides angular stability in terms of incident angles in the frequency response of the structure. The FSMS element has dimensions of 0.3*λ_o_* × 0.3*λ_o_* × 0.05*λ_o_* and is designed and simulated in a commercially available full-wave simulator (Ansys HFSS).

### FSMS Unit Cell Rotation at 45°

The FSMS element is rotated at an angle of 45° to verify the polarization behavior as depicted in Figure 1b. The proposed design manifests polarization-independent behavior. The FSMS element rotated at 45° demonstrates similar EM performance to its un-rotated counterpart. The shielding characteristics of the FSMS element rotated at 45° for both the transverse electric and transverse magnetic modes of polarization are presented in angular stability analysis section. It may be observed that the notch bandwidth increases with an increment in the incident angle. However, minor variations in the frequency response are also observed as the incident angle increases. The notch selectivity at resonant frequency also improves over incident angle variations. Moreover, Figure 1c demonstrates the working principal of the anticipated FSMS.

## 3. Simulations, Results, and Discussions

**Figure 2:**
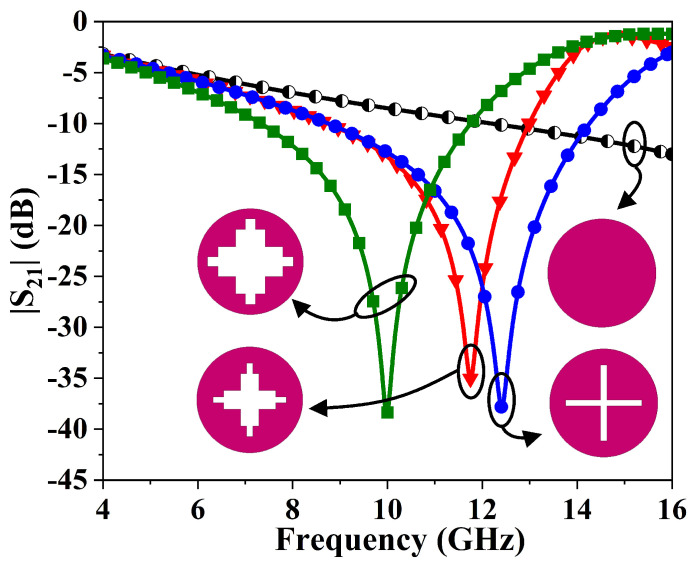
Step-by-step design process of the FSMS element and its response at normal incidence.

This section describes simulation of the stopband FSMS and optimization of its spectral response. Initially, a circular metallic patch with a diameter of L+2d is implemented, as shown in Figure 2. Next, a rectangular strip with an area of L×S, along with its replica on the y-axis, is subtracted from the center of the patch. The resulting structure resonates at a frequency of 12.5 GHz, as illustrated in Figure 2. Moreover, a second slot with an area of m×n is etched along the x- and y-axes to achieve structural symmetry and a dual-polarized response of the FSMS. Consequently, the structure resonates at about 11.8 GHz. Further tuning of the dimensions of the engraved slots lowers the resonant frequency. Finally, the optimized geometry achieves stopband characteristics at 10 GHz X-band central frequency.

### 3.1. Shielding Effectiveness

The shielding effectiveness (SE) quantifies the ability of an FSMS filter to attenuate electromagnetic waves across specific frequency ranges. A higher value of the SE indicates better shielding performance. Mathematically, SE is often expressed in decibels (dB) as follows [1,34,37]:

(1) $$\mathrm{SE}\left(\mathrm{dB}\right)=-20\log_{10}\left|\frac{E_{t}}{E_{i}}\right|,$$

where $E_{t}$ and $E_{i}$ denote the electric fields transmitted through and incident on the FSMS surface, respectively. The SE can be further expressed in terms of $|S_{21}|$ as follows [1,34,37]:

(2) $$\mathrm{SE}\left(\mathrm{dB}\right)=-20\log_{10}\left|S_{21}\right|.$$

### 3.2. Angular Stability Analysis

**Figure 3:**
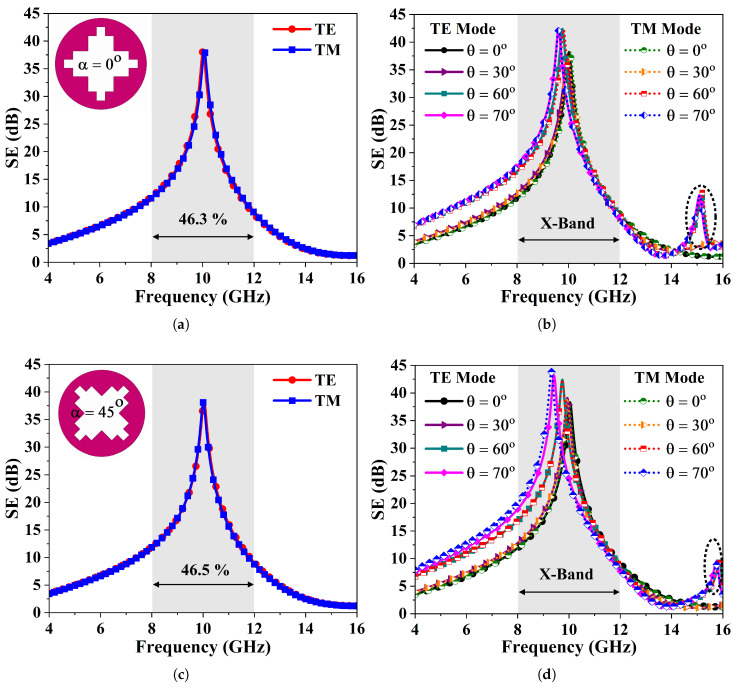
The SE of un-rotated FSMS element under TE and TM polarizations (**a**) at normal incidence, (**b**) at oblique angles up to 70°. (**c**) SE of FSMS rotated at 45° at *θ* = 0° for TE and TM wave modes. (**d**) FSMS’s SE as a function of oblique angle (*θ* > 0°) for the TE and TM polarization states.

The designed FSMS is exposed to various incident oblique angles and polarization states to thoroughly analyze its electromagnetic behavior. Figure 3a presents the shielding characteristics of the FSMS filter for TE and TM wave modes at normal incidence (*θ* = 0°). It has been observed that the FSMS reveals similar spectral responses for both polarization modes while achieving an effective shielding of 38.5 dB at the resonant frequency. It offers a 10 dB fractional suppression bandwidth of 46.3% at 10 GHz frequency. The incident angle is varied from 0° to 60° with a step size of 30°. Figure 3b depicts the SE versus frequency curves of the metasurface over different oblique incidences under TE and TM polarizations. It can be noticed that the SE and rejection bandwidth improve at the resonant frequency, as the incident angle varies, while the design exhibits identical and stable frequency response over all the incident angles. A relative deviation of 2.15% in the transmission zero frequency is noted at 70°. A grating lobe appears at about 15.25 GHz when the incident angle approaches 60° and 70°; however, it is out of the desired band and does not affect performance in the band of interest.

Moreover, the FSMS element is rotated at an angle of 45° to verify its polarization behavior. The proposed design reveals polarization-independent behavior. The FSMS element rotated at 45° exhibits the same performance to its un-rotated counterpart. The shielding response of the FSMS element rotated at 45° for the TE and TM modes of the polarization at normal incidence are presented in Figure 3c. It is obvious from the figure that FSMS obtains a SE of at least 39 dB and effectively suppresses X-band signals. At *θ* = 0°, FSMS offers a fractional stop-bandwidth of 46.5% for TE- and TM-polarized waves. In addition, Figure 3d highlights the FSMS’s SE as a function of incident angle up to 70° for the TE and TM wave modes. The notch selectivity and rejection bandwidth at resonant frequency improve with larger oblique angles. The additional resonance occurring at 15.25 GHz is shifted towards the higher frequencies and its magnitude is also reduced. However, a minor variation of 2.96% in the frequency response of the rotated FSMS is observed as the incident angle (*θ*) increases from 0° to 70° which can be computed as follows [30,37]:

(3) $$\Delta f=\left|\frac{f_{z}-f_{\mathrm{oblique}}}{f_{z}}\right|\times 100,$$

where Δ*f*, $f_{z}$, and $f_{\mathrm{oblique}}$ stands for frequency deviation, transmission zero frequency at *θ* = 0°, and oblique angle (*θ* > 0°) frequency, respectively. These variations are associated with change in surface impedance with incident angles, which can be calculated as follows [30,37]:

(4) $$Z_{\mathrm{TE}}^{\mathrm{FSMS}}=\frac{Z_{{}^{\circ}}}{\cos\theta },$$

(5) $$Z_{\mathrm{TM}}^{\mathrm{FSMS}}=Z_{{}^{\circ}}\cos\theta ,$$

where $Z_{\mathrm{TE}}$ and $Z_{\mathrm{TM}}$ represent the corresponding impedances perceived by incident TE- and TM-polarized waves. $Z_{{}^{\circ}}$ and θ are air’s impedance and incident angle of EM waves, respectively. The overall performance of the FSMS in both configurations demonstrates that the design ensures polarization- and rotation-independent behavior, owing to its miniaturized fourfold structural symmetry. In addition, FSMS’s periodicity (P) strongly influence its angular performance. Therefore, to ensure high angular stability and prevent early onset of the grating lobes, the limiting value of *P* can be obtained through [2]

(6) $$P<\frac{\lambda_{o}}{1+\sin(\theta )},$$

where $\lambda_{o}$ is free-space wavelength at the notching frequency and θ refers angle of incidence. In this particular instance at angle (*θ* = 70°), the limited periodicity value is P=15.46 mm. For the proposed FSMS design the cell periodicity is 9 mm, which is less than the limiting value. Therefore, the FSMS exhibits excellent angular stability and polarization insensitivity for both design configurations.

### 3.3. Surface Current Distribution

**Figure 4:**
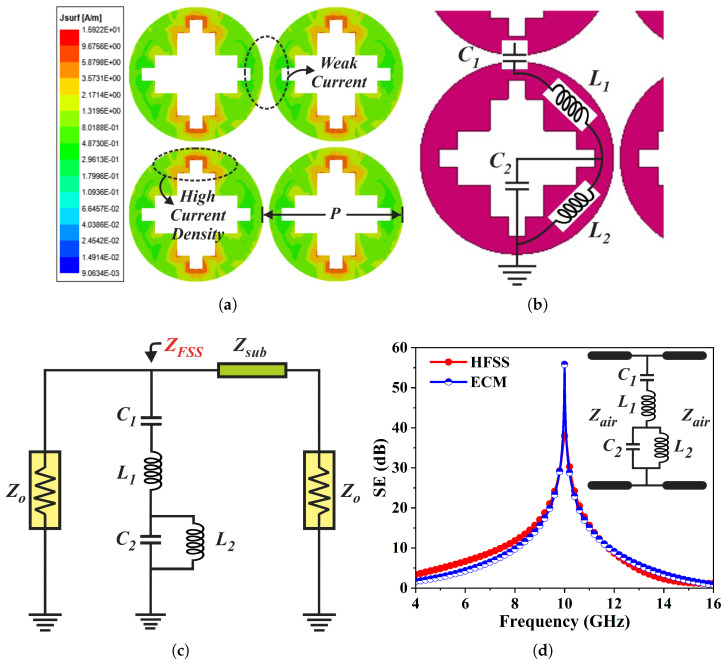
(**a**) Surface current density plot of a 2 × 2 FSMS array at 10 GHz frequency for TE-polarized wave at normal incidence. (**b**) Lumped element formation to design an ECM of the FSMS element. (**c**) Derived circuit model of FSMS with optimized lumped parameters as $C_{1}=0.91$ pF, $C_{2}=0.71$ pF, $L_{1}=0.12$ nH, and $L_{2}=0.11$ nH. (**d**) SE comparison of the equivalent circuit model (ECM) versus EM simulation (HFSS).

Figure 4a illustrates the surface current density (J-surf) of the FSMS array examined at its resonant frequency, under normal incidence (TEz-0°) for the TE wave mode. The current distribution manifests that the induced surface currents are more prominent along the vertical edges of the FSMS surface, suggesting that these regions are responsible for resonance at the operating frequency. The loops or closed sections in the geometry signify an inductive effect, whereas the gaps or slots in the structure imply a capacitive effect. The current flow paths can be controlled simply by varying length and width of the staircase slot instead of varying the diameter of metallic patch.

### 3.4. Equivalent Circuit Model of FSMS Element

The derivation of an equivalent circuit model (ECM) of an FSMS is primarily dependent on its geometry and polarization of incident electromagnetic waves [38]. The surface current distribution plot in Figure 4a clues to the formation of inductive and capacitive components. The strong current intensity at the vertical edges of the circular patch contributes to inductance, while the gaps come up as capacitance. Figure 4b specifies the formation of lumped elements. The capacitances $C_{1}$ and $C_{2}$ are formed at the inter-element spacing and the staircase slot, whereas the inductance $L_{1}$ and $L_{2}$ are formed at the metallic edges of the circular patch. Thus, a derived ECM of the FSMS structure is depicted in Figure 4c. It can be observed that $C_{1}$ and $L_{1}$ are connected in series while $C_{2}$ and $L_{2}$ make a parallel combination. Moreover, $Z_{0}$ is the characteristic impedance ($Z_{0}=377\;\Omega$) of free space on both sides of the FSMS. The substrate is modeled as a transmission line having a characteristic impedance of $Z_{\mathrm{sub}}=\frac{Z_{0}}{\sqrt{\epsilon_{r}}}$. The ECM is designed and simulated in agilent advanced design system (ADS). The analysis reveals that the ECM attains a stopband at 10 GHz while its impedance can be expressed as

(7) $$Z_{\mathrm{FSS}}=\left(j\omega L_{1}+\frac{1}{j\omega C_{1}}\right)+\left(\frac{1}{\frac{1}{j\omega L_{2}}+j\omega C_{2}}\right),$$

(8) $$Z_{\mathrm{FSS}}=\frac{\left[\omega^{2}C_{1}L_{2}-\left(1-\omega^{2}C_{1}L_{1}\right)\left(1-\omega^{2}C_{2}L_{2}\right)\right]}{j\omega C_{1}\left(1-\omega^{2}C_{2}L_{2}\right)},$$

where ω is the angular frequency. The increment in $C_{1}$ and $L_{1}$ lowers the resonant frequency. The derived expression can be classified into two zeros and two poles. The lumped parameters are determined by setting both the numerator and denominator of (8) to zero and solving the resulting equations.

(9) $$C_{2}=\frac{1}{L_{2}\omega_{p2}^{2}},$$

(10) $$C_{1}=\frac{\left(\omega_{p2}^{2}-\omega_{z1}^{2}\right)\left(\omega_{p2}^{2}-\omega_{z2}^{2}\right)}{-L_{2}\omega_{p2}^{2}\omega_{z1}^{2}\omega_{z2}^{2}},$$

(11) $$L_{1}=\frac{\left(\omega_{p2}^{2}-\omega_{z1}^{2}\right)-C_{1}L_{2}\omega_{p2}^{2}\omega_{z1}^{2}}{C_{1}\omega_{z1}^{2}\left(\omega_{p2}^{2}-\omega_{z1}^{2}\right)},$$

In the above expressions, $\omega_{p2}$, $\omega_{z1}$, and $\omega_{z2}$ represent the pole and the two zeros, while the first pole $\omega_{p1}$ is zero. The remaining lumped parameters are computed by iterative insertion of $L_{2}$ value. Figure 4d highlights a comparison of the SE curves of the circuit and the EM models. It can be observed from the figure that the frequency response of the ECM coincides well with one obtained from the HFSS. In addition, lumped element values can be calculated based on the FSMS’s physical dimensions [38,39,40]. The inductances and capacitances are computed through (12) and (13), respectively.

(12) $$L=\mu_{0}\frac{b}{2\pi }\ln\left(\frac{1}{\sin\frac{\pi w}{2b}}\right),$$

(13) $$C=\varepsilon_{0}\varepsilon_{\mathrm{eff}}\frac{2P}{\pi }\ln\left(\frac{1}{\sin\frac{\pi m}{2P}}\right),$$

where *b* and *w* are the length and width of metallic stubs contributing to inductance, *P* is the length of strips imparting capacitances, and *m* is the inter-element spacing in an array. Furthermore, the transmission coefficient $|S_{21}|$ of the ECM may alternatively be computed using transmission line theory (14) following evaluation of the lumped parameters [41,42].

(14) $$S_{21}=\frac{2Z_{0}}{AZ_{0}+B+CZ_{0}^{2}+DZ_{0}},$$

where A, B, C, and D are coefficients of the ABCD matrix of the system consisting of FSMS with N dielectric layers.

(15) $$\left[\begin{array}{cc} A & B\\ C & D \end{array}\right]=\left[M_{\mathrm{FSMS}}\right]\left[M_{n+1}\right]\cdots \left[M_{N}\right],$$

In (15), $M_{n}$ refers to the scattering matrix of nth dielectric substrate. Since the proposed FSMS is a single-layer design, the expression in (15) is reduced to (16).

(16) $$\left[\begin{array}{cc} A & B\\ C & D \end{array}\right]=\left[M_{\mathrm{FSMS}}\right]\left[M_{1}\right],$$

(17) $$\left[M_{1}\right]=\left[\begin{array}{cc} \cos(k_{z1}d) & jZ_{0}\sin\left(k_{z1}d\right)\\ \frac{j\sin(k_{z1}d)}{Z_{0}} & \cos(k_{z1}d) \end{array}\right],$$

(18) $$\left[M_{\mathrm{FSMS}}\right]=\left[\begin{array}{cc} 1 & 0\\ \frac{1}{Z_{\mathrm{FSMS}}} & 1 \end{array}\right],$$

The similar approach can be applied for solving the TE and TM wave modes, substituting $Z_{\mathrm{FSMS}}$ in (18) with $Z^{\mathrm{TE}}$ and $Z^{\mathrm{TM}}$ as specified in the subsequent expression, respectively.

(19) $$Z^{\mathrm{TE}}=\frac{\omega \mu_{r}\mu_{0}}{k_{z1}},\;Z^{\mathrm{TM}}=\frac{k_{z1}}{\omega \varepsilon_{r}\varepsilon_{0}},$$

where $k_{z1}$ and $k_{t}$ denote the normal and tangential components of the wavenumber as expressed in (20) and (21), respectively. While $\mu_{r}$$\mu_{0}$ and $\varepsilon_{r}$$\varepsilon_{0}$ are the relative permeabilities and relative permittivities of free-space and dielectric, respectively. The thickness of dielectric is *d*, $k_{0}$ is the free-space propagation constant, and θ represents angle of incidence of EM wave-striking FSMS surface.

(20) $$k_{z1}=\sqrt{\varepsilon_{r}k_{0}^{2}-k_{t}^{2}},$$

(21) $$k_{t}=k_{0}\sin\theta ,$$

The impedance of FSMS structure under various oblique incident angles and polarizations (TE and TM) of the striking EM waves can be expressed as in [41].

(22) $$\left[\begin{array}{cc} Z_{\mathrm{TE},\mathrm{TE}}^{\mathrm{FSMS}}\left(k_{x},k_{y}\right)\; & Z_{\mathrm{TM},\mathrm{TE}}^{\mathrm{FSMS}}\left(k_{x},k_{y}\right)\\ Z_{\mathrm{TE},\mathrm{TM}}^{\mathrm{FSMS}}\left(k_{x},k_{y}\right)\; & Z_{\mathrm{TM},\mathrm{TM}}^{\mathrm{FSMS}}\left(k_{x},k_{y}\right) \end{array}\right],$$

where $k_{x}$ and $k_{y}$ represent the transverse wavenumbers associated with the oblique incident angle given as $k_{x}=k_{0}\sin\left(\theta \right)\cos\left(\varphi \right)$ and $k_{y}=k_{0}\sin\left(\theta \right)\sin\left(\varphi \right)$. In fourfold symmetric FSMS unit cells, the off-diagonal terms, due to modal coupling effects and azimuthal angle (ϕ), are negligible compared to the dependence angle (θ). Thus, the oblique incidence impedance of the FSMS is only a two-terms diagonal matrix.

## 4. Parametric Analysis

### Geometric Optimization

**Figure 5:**
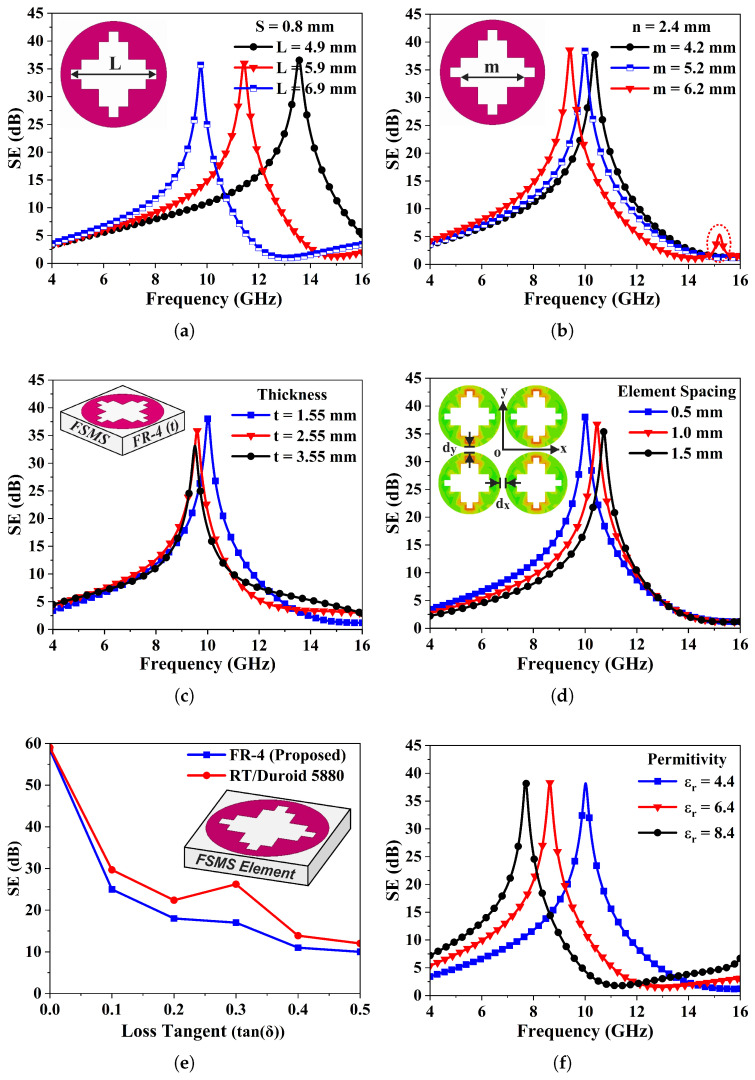
(**a**) Variationsin length of middle slot of the staircase slot to optimize FSMS’s frequency response. (**b**) Effect of change in length of accompanying slots on FSMS element performance while keeping its width fixed. (**c**) Impact of variations in dielectric thickness on FSMS filtering characteristics. (**d**) SE versus frequency curves of the FSMS element as a function of inter-element spacing. (**e**) The FSMS element’s SE analysis as a function of dielectric loss (tan(δ)). (**f**) Influence of dielectric permittivity on the FSMS EM shielding characteristics.

A comprehensive parametric analysis is performed to establish a correlation between various geometrical parameters and frequency response of the anticipated design. The dimensions of staircase slot are varied to study its impact on the resonant frequency and the shielding performance of the FSMS. Figure 5a signifies the effect of varying the length (*L*) of middle slot of the staircase profile, keeping its width fixed. It is noted that the increase in the length of the slot significantly lowers the resonant frequency and vice versa.

Likewise, Figure 5b illustrates the effects of change in length (*m*) of the accompanying slots located above and below the middle slot, while keeping its width fixed. The figure shows that the notching frequency shifts upward with decrease in length, yet it has a minimal impact on resonant frequency or shielding effectiveness. However, these variations significantly contribute for enhancing the angular stability and operating bandwidth of the FSMS. Due to the design’s symmetry along the x- and y-axis, it exhibits polarization-insensitive spectral characteristics across different incident angles. Moreover, Figure 5c investigates the impact of laminate thickness on FSMS frequency response, showing that decreasing the thickness shifts resonant frequency towards higher frequencies and also enhances the stop-bandwidth, along with improvement in SE. The results in Figure 5d indicate that smaller inter-element spacing enhances angular stability, shifts the resonant frequency downward, and improves both the SE and rejection bandwidth.

In addition, Figure 5e compares FSMS’s shielding performance with a low-loss substrate to strengthen its practical applicability. The figure illustrates that SE steadily declines as dielectric loss increases. However, for low-loss substrate, the design offers superior SE. Figure 5f reveals FSMS’s performance as a function of dielectric permittivity ($\varepsilon_{r}$). It is observed from the figure that an increase in dielectric constant results in lowering of the resonant frequency and reduction of the suppression bandwidth, whereas the SE of FSMS element remains unchanged.

## 5. Prototype Fabrication and Experimental Verification

### 5.1. Measurement Setup

**Figure 6:**
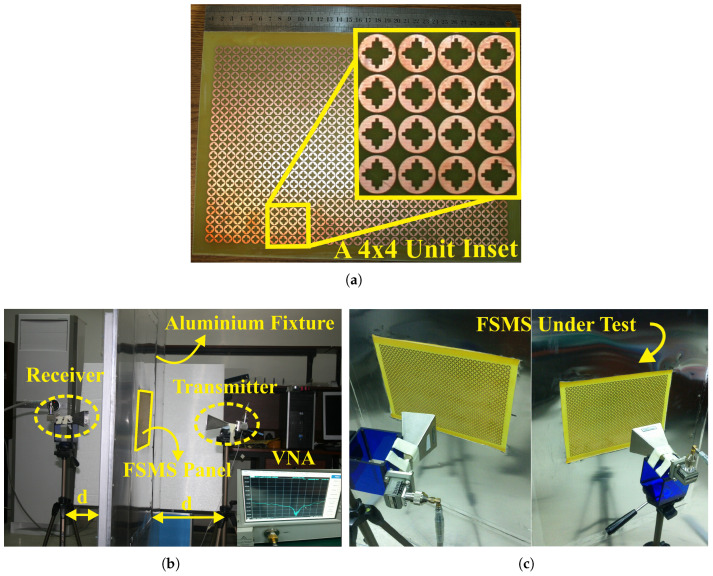
(**a**) A photograph of fabricated prototype with a zoomed-in view provided as an inset. (**b**) Measurement setup for the experimental validation of fabricated FSMS. (**c**) FSMS under test to validate its angular performance.

To validate the simulated design, a prototype is fabricated and measured using the technique proposed in [43,44]. A 6 × 4 feet aluminum wall with an aperture in the middle was used as a test jig. A thorough calibration without an FSMS sample was performed prior to measurement to cater for various losses including cable losses, diffraction losses from the edges, etc. A rectangular grid of 33 × 22 FSMS unit elements was patterned on an FR-4 substrate as shown in Figure 6a. Agilent N5242A PNA was used to measure the transmission coefficient of the fabricated FSMS panel. A pair of Lucas-Nuelle® WR90 horn antennas were used for X-band measurements as depicted in Figure 6b. These antennas have 15 dBi gain for frequency ranges of 8 to 12 GHz. The antennas are connected to the ports of the PNA. One antenna is used as a transmitter (Tx), and the other acts as a receiver (Rx). These antennas are kept at far-field distance from the FSMS panel. The far-field distance is formed as $d\geq \frac{2D^{2}}{\lambda }$, where *D* denotes horn antenna size and λ is the wavelength at FSMS’s operating frequency. Additionally, Figure 6c signifies the measurements carried out to validate the oblique incident angle performance under TM and TE polarizations.

### 5.2. Measured Results

**Figure 7:**
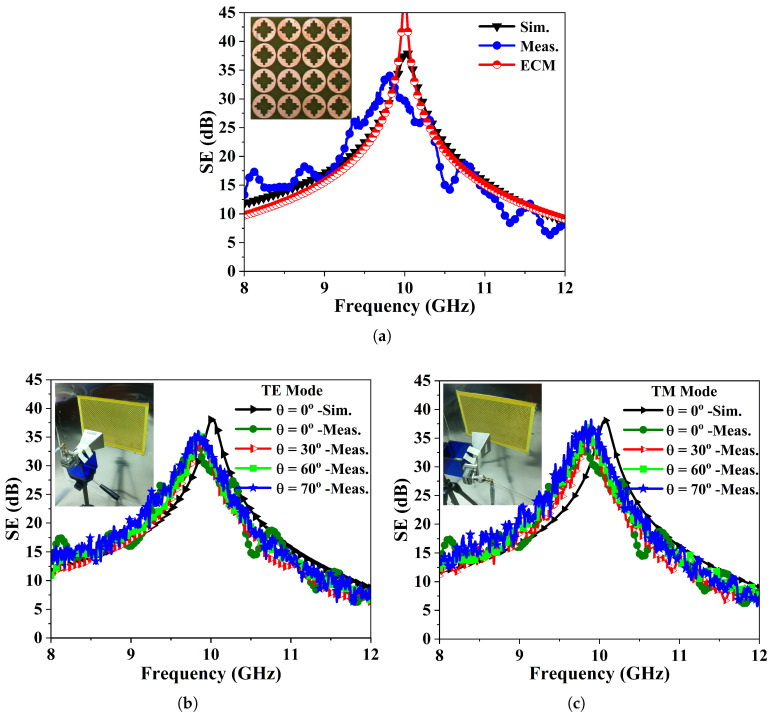
(**a**) A SE comparison of the FSMS obtained through simulation, measurement, and the equivalent circuit model. (**b**) TE wave mode shielding characteristics of the FSMS array as a function of oblique angle. (**c**) SE of the fabricated FSMS panel at oblique incidences ($\theta >0^{{}^{\circ}}$) under TM wave mode ensuring wide angular stability.

Figure 7a illustrates a comparison of the shielding effectiveness (SE) of the anticipated metasurface (FSMS) obtained through the EM simulation, measurements, and the equivalent circuit model. It can be noticed that the results have significant correlation. The measured SE of the fabricated FSMS panel for the TE and TM polarization states are plotted in Figure 7b and Figure 7c, respectively. The incident angle is varied from 0° to 70°. The proposed FSMS reveals a stable spectral responses at various oblique angles for both the TE and TM polarization modes. The FSMS exhibits measured SE of not less than 35 dB at the resonant frequency under various angles. It is noticed that the peak transmission zero frequency is shifted downward by approximately <2.37% for both TE and TM polarizations. These variations in the measured results are due to the losses associated with RF-Cables, fabrication tolerances, the FR-4 substrate, and measurement imperfections.

## 6. Comparison with Recent Literature

Table 1 provides a summary of the reviewed FSS designs, comparing metrics of the proposed FSMS with those reported in the recent literature. The comparison highlights that most of the related works offer good shielding levels and wider angular stability while maintaining miniaturized dimensions, but they also have performance limitations, i.e., narrower suppression bandwidth and reduced shielding effectiveness over angle variations, particularly for the TM polarization mode. For instance, studies in [17,20,27], despite having miniaturized dimensions compared to the presented FSMS, their SE and stop-bandwidth continuously decline with variations in incident angle for the TM mode. References [24,27,35] have large cell size, leading to limited angular performance and TM mode instability in terms of SE and bandwidth. However, [24] manifests polarization-independent characteristics. In addition, FSS reported in [23] have polarization-insensitive and angularly stable spectral response but offers narrower stop-bandwidth. In [45], the FSS has limited angle stability. In contrast, anticipated FSMS mitigates the performance degradation issue in the TM mode and outperforms rest of the reported designs. It exhibits consistent angularly stable and polarization-insensitive spectral responses across varying incident angles up to 70°, which is attributed to its fourfold structural symmetry and compact size, making it a strong contender for selective EMI shielding. Additionally, the FSMS is also scalable to other frequencies. It can bear fabrication tolerances up to 0.25 mm for the staircase-slot dimensions. However, fabrication imperfections for the circular patch will effect the inter-element spacing causing frequency shifts.

## 7. Conclusions

In this paper, a miniaturized frequency-selective metasurface (FSMS) is proposed for band-notch applications. The FSMS features a circular patch with a staircase slot, enabling unit cell miniaturization and frequency stability at oblique incidences. The FSMS notches at 10 GHz X-band frequency and achieves a measured SE of 35 dB. It manifests identical transmission responses for TE and TM wave modes up to 70°, ensuring high angular stability and polarization independence. The FSMS element is also rotation-independent. A prototype FSMS panel of 33 × 22 unit cells is fabricated and tested for validation of the anticipated design. In addition, the simulated and measured results are in good agreement. The results indicate that the proposed design is effective for EMI suppression and can be employed in the development of 5G infrastructure and military applications.

## Figures and Tables

**Table 1:** A comparisonof this FSMS with recently reported literature for EM shielding applications.

Ref. No.	Unit Cell Size	Substrate Material	Operating Bands	SE (dB)	10 dB FBW (%)	Angle Stability	TM Mode Stability	Polarization-Insensitive
[19]	0.05$\lambda_{o}$ × 0.05$\lambda_{o}$ × 0.006$\lambda_{o}$	FR-4	WLAN	38.8	53	$75^{{}^{\circ}}$	N/A	Yes
[20]	0.09$\lambda_{o}$ × 0.09$\lambda_{o}$ × 0.007$\lambda_{o}$	FR-4	WLAN	34	N/A	$60^{{}^{\circ}}$	N/A	Yes
[23]	0.20$\lambda_{o}$ × 0.20$\lambda_{o}$ × 0.05$\lambda_{o}$	FR-4	X-Band	33	26 and 31	$60^{{}^{\circ}}$	Yes	Yes
[24]	0.31$\lambda_{o}$ × 0.31$\lambda_{o}$ × 0.002$\lambda_{o}$	Polyester	WLAN	32	25	$45^{{}^{\circ}}$	N/A	Yes
[27]	0.45$\lambda_{o}$ × 0.45$\lambda_{o}$ × 0.05$\lambda_{o}$	FR-4	X-Band	34.8	44	$45^{{}^{\circ}}$	N/A	N/A
[28]	0.24$\lambda_{o}$ × 0.24$\lambda_{o}$ × 0.004$\lambda_{o}$	Rogers 5880	X-band	30	32	$60^{{}^{\circ}}$	N/A	N/A
[35]	0.46$\lambda_{o}$ × 0.46$\lambda_{o}$ × 0.23$\lambda_{o}$	FR-4	5G NR	30	7.1	$40^{{}^{\circ}}$	N/A	N/A
[45]	0.15$\lambda_{o}$ × 0.15$\lambda_{o}$ × 0.053$\lambda_{o}$	FR-4	X-Band	35	40	$45^{{}^{\circ}}$	Yes	Yes
This Work	0.30$\lambda_{o}$ × 0.30$\lambda_{o}$ × 0.05$\lambda_{o}$	FR-4	X-Band	38.1/35.2	46.3 and 46.5	$70^{{}^{\circ}}$	Yes	Yes

$\lambda_{o}$ is the free-space wavelength at the first resonant frequency. N/A: Not Appreciable.

## Data Availability

Data are contained within the article.
